# Achieving Crossed Strong Barrier Coverage in Wireless Sensor Network

**DOI:** 10.3390/s18020534

**Published:** 2018-02-10

**Authors:** Ruisong Han, Wei Yang, Li Zhang

**Affiliations:** 1 School of Electronic and Information Engineering, Beijing Jiaotong University, Beijing 100044, China; hanruisong@bjtu.edu.cn; 2 School of Electronic and Electrical Engineering, University of Leeds, Leeds LS2 9DX, UK; l.x.zhang@leeds.ac.uk

**Keywords:** barrier coverage, crossed, wireless sensor networks, branch and bound, shortest path

## Abstract

Barrier coverage has been widely used to detect intrusions in wireless sensor networks (WSNs). It can fulfill the monitoring task while extending the lifetime of the network. Though barrier coverage in WSNs has been intensively studied in recent years, previous research failed to consider the problem of intrusion in transversal directions. If an intruder knows the deployment configuration of sensor nodes, then there is a high probability that it may traverse the whole target region from particular directions, without being detected. In this paper, we introduce the concept of crossed barrier coverage that can overcome this defect. We prove that the problem of finding the maximum number of crossed barriers is NP-hard and integer linear programming (ILP) is used to formulate the optimization problem. The branch-and-bound algorithm is adopted to determine the maximum number of crossed barriers. In addition, we also propose a multi-round shortest path algorithm (MSPA) to solve the optimization problem, which works heuristically to guarantee efficiency while maintaining near-optimal solutions. Several conventional algorithms for finding the maximum number of disjoint strong barriers are also modified to solve the crossed barrier problem and for the purpose of comparison. Extensive simulation studies demonstrate the effectiveness of MSPA.

## 1. Introduction

Wireless sensor networks (WSNs), which have the outstanding advantages of easy configuration, flexibility in shrinking or expanding, strong fault-tolerance, and mobility, have played an important role in monitoring and analyzing dynamic, hostile, unfamiliar, and unexplored environments. In some monitoring and early warning applications, such as border protection, battlefield surveillance, and animal migration observation, the primary objective is to detect the intruders who penetrate the target regions with sensor nodes. In WSNs, a series of sensor nodes, whose sensing regions overlap, form a sensor barrier for intruders and can guarantee the detection of penetrating behavior in particular directions. Thus, designing strategies for forming sensor barriers has great importance and is often referred to as the barrier coverage problem [1,2,3,4].

In contrast to the full coverage presented in [5,6], which requires detecting intruders at every point in their trajectory, barrier coverage only ensures that the intrusion behavior is detected. The main concerns of the two coverage problems in coverage intensity and the movement of monitoring targets are different. Thus, with a lower number of sensor nodes, barrier coverage still achieves a satisfactory level of intruder detection.

**Figure 1:**
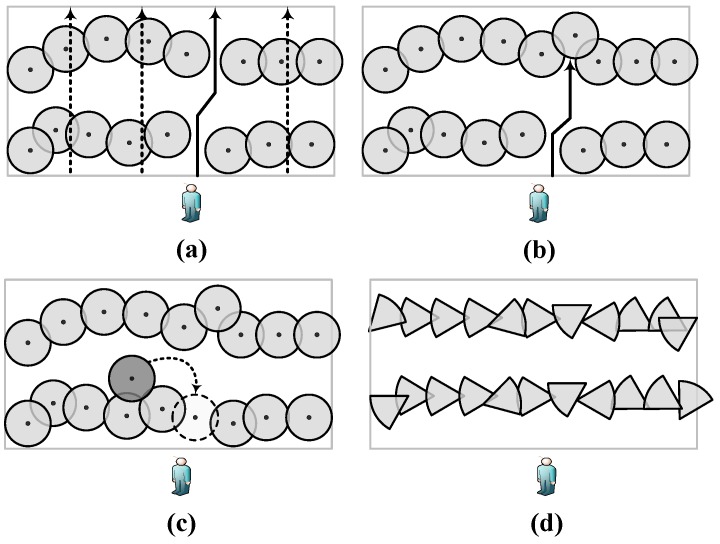
Various types of barrier coverage in wireless sensor networks (WSNs): (**a**) Weak barrier coverage. (**b**) Strong barrier coverage. (**c**) Hybrid barrier coverage. (**d**) Barrier coverage in directional sensor networks (DSNs).

Basically, barrier coverage can be classified into weak barrier coverage and strong barrier coverage [1]. In weak barrier coverage, the horizontal projections of sensing regions overlap, only guaranteeing to detect movements along vertical traversing paths, as illustrated by the dash lines in Figure 1a. Meanwhile, as shown in the same figure, if an intruder knows where the sensor nodes are, it may adopt a polygonal path, indicated by the solid line, without being detected. In contrast, strong barrier coverage, which provides continuous coverage, ensures that every intrusion is detected since any crossing path needs to traverse a barrier. As shown in Figure 1b, despite following a polygonal path, the intruder can be detected by the strong barrier on the top.

Additionally, based on the characteristics of barriers, barrier coverage can be classified in other ways: local barrier coverage and global barrier coverage, 1-barriers and *k*-barriers, coverage with mobile sensors and coverage with stationary sensors, etc. As illustrated by Figure 1c, if a sensor with mobility (indicated by the dark circle) is used and assigned to move to the position indicated by the dash lines, it would form a 2-barrier coverage in the hybrid WSN, which employs both mobile and stationary sensors. Moreover, with the rapid development of directional sensor networks (DSNs), barrier coverage with directional sensing models (shown in Figure 1d) has attracted a great deal of interest.

Unfortunately, although strong barrier coverage can detect intruders who traverse the target region vertically, a security vulnerability exists since a WSN cannot handle the traverse intrusion. For the application of observing animal migration or battlefields, it is also of great importance to detect traversing targets since it helps us to grasp the motion law of intruders and develop strategies to enhance the security level of a system.

When using previous research results to design and configure a sensor barrier, people are incapable of detecting intrusion behavior in both horizontal and vertical directions. Thus, we need to find a series of sensor nodes, whose sensing regions overlap and can form two continuous paths that link the boundaries of the horizontal and vertical directions. In this paper, that series of sensor nodes is referred to as a crossed strong barrier (crossed barrier for short). In brief, crossed barrier coverage can increase the possibility of detecting intrusion and thus improve system security.

Thus, we introduce the concept of crossed barrier coverage, which consists in the detection of both horizontal and vertical intrusions, to tackle the challenges mentioned above. Crossed barrier coverage applies to more complex situations where the conventional barrier cannot satisfy the security requirements of a system. Moreover, as argued in [1,7], scheduling sensor nodes to work in barriers leads to both savings in node numbers and extensions in network lifetime. The savings are achieved since redundant sensor nodes can go into sleep mode or be removed. Thus, we attempt to determine the maximum number of crossed barriers in order to extend the lifetime of a WSN. Note that maximizing the number of crossed barrier is not merely the superposition of finding and adding up the maximum barrier in individual directions. We demonstrate it through analysis and simulations in the following sections. The main contributions of our work are described as follows:

1. To the best of our knowledge, we are the first to introduce and study crossed barrier coverage in WSNs. We also show that the problem of finding the maximum number of crossed strong barriers is NP-hard.
2. We provide an integer linear programming (ILP) formulation to better describe the optimization problem of finding the maximum number of crossed barriers, and we use the branch-and-bound algorithm to obtain the optimal solution, which will serve as a benchmark for other algorithms.
3. We propose a heuristic algorithm called a multi-round shortest path algorithm (MSPA) to find the maximum number of crossed barriers. The MSPA can achieve near-optimal solutions in polynomial time.
4. We modified the algorithms used in the conventional barrier coverage problem to make them suitable for the new problem and conducted extensive simulations to evaluate their performance.

The remainder of this paper is organized as follows. We provide a brief discussion about related literature and work in Section 2. Section 3 introduces the system model and the problem statement. In Section 4, we present the ILP formulation for the optimization problem and solve it using the branch-and-bound algorithm. In Section 5, we present our MSPA method and several modified algorithms. Simulation work and numerical results are presented in Section 6, and we conclude the paper in Section 7.

## 2. Related Work

The concept of barrier coverage was originally proposed in the context of robotics sensors [8] and first introduced into WSNs in [1]. Since then, active research has been carried out in this area in the following aspects. Note that we summarized these research works not only to provide the preliminary knowledge about barrier coverage, but also to indicate further research directions for the crossed barrier coverage problem.

**Sensing model:** In conventional studies of the barrier coverage problem [9,10,11,12,13,14], the Boolean disc sensing model [15], which is expressed by an omni-directional circle, is widely used. In this model, a target inside or outside the sensing range of a sensor node is detected by the sensor with probability one or zero. Although it is simple for analysis, the model cannot fully characterize the sensor measurements, which are usually affected by noise and vary with the distance between the sensor and the target. Thus, the work in [16,17,18] assumed the probabilistic sensing model. Moreover, state-of-the-art research work considers more practical issues such as the utilization of the three-dimensional (3D) sensing model in camera sensor networks [19] and the joint probability model in the compound event barrier coverage problem [20]. However, we adopt the Boolean disc sensing model in our research since we are the first to study the crossed barrier coverage problem.

**Deployment method:** There are two main approaches to barrier coverage in WSN: random deployment and deterministic deployment [21]. The former involves scheduling randomly distributed sensor nodes in a WSN to build sensor barriers, while the latter aims at globally optimizing the locations of sensors to minimize the total number of sensors, while ensuring the performance of barrier coverage. For deterministic deployment, based on the geometric shape, the curve-based deployment and line-based deployment are respectively presented in [21,22,23,24]. We analyzed the scenario with random deployment since it is more common for large-scale WSNs.

**Coverage intensity:** Coverage intensity is measured by the barrier number, the continuity of barriers, and the probability of detecting a target. Based on the coverage intensity that a WSN provides, barrier coverage is categorized in the following ways: strong barrier coverage and weak barrier coverage, 1-barriers and k-barriers, and worst- and best-case coverage and exposure path coverage [15]. Here, strong barrier coverage is studied, and barrier number is adopted as the measurement of coverage intensity.

**Sensor mobility:** Deploying mobile sensors in a WSN can be extremely valuable in hostile environments and has recently attracted great interest. Barrier coverage with the consideration of sensor mobility [25,26,27,28,29] aims at efficiently improving barrier coverage under the constraints of the available mobile sensors and their moving range. For example, in [28,29], the authors tried to efficiently form barrier coverage by leveraging mobile sensors to fill in the gaps between pre-deployed stationary sensors, while ensuring that the number or the total moving cost of mobile sensors is at a minimum. Nonetheless, sensor mobility is not considered in this paper since we aim at solving the barrier coverage problem by making the most of stationary sensors.

**Network lifetime:** The initial aim of introducing barrier coverage was to maintain a certain intruder-detecting capacity and extend the lifetime of the whole WSN. In [7], a wakeup schedule for individual nodes was proposed to maximize network lifetime, utilizing the redundancy of sensors. In [30], the authors focus on the effect of the lifetime of sensor nodes and prove that the heterogeneity of sensor lifetime affects the barrier number. Thus, to achieve more efficient barrier coverage in practice, lifetime issues should be considered in future research.

As mentioned in the previous section, the traversal intrusion is a security vulnerability for strong barrier coverage. Based on the above-mentioned aspects, it can be seen as a coverage intensity problem. Meanwhile, maximizing the number of crossed barriers helps to extend the lifetime of the WSN. The following literature provides a rudimentary knowledge of the work related to ours.

In [1], Kumar et al. demonstrated how to use a centralized method to find disjoint barriers. The core idea is to use the maximum flow algorithm in graph theory, and this algorithm is widely used in research on the strong barrier coverage problem. In [31], the authors present heuristic ways to eliminate strong barriers that have conflicts, but the barrier coverage problem they solved is limited to the horizontal direction.

In [13], which inspires our research most, Liu et al. derived critical conditions for strong barrier coverage and devised an efficient distributed algorithm to construct disjoint barriers. In their algorithm, they constructed vertical barriers in vertical strips to connect the horizontal barriers in the adjacent segments. Vertical barriers help to prevent intruders from moving between segments, resulting in a more robust network. However, the authors did not provide further details on how to choose a single barrier by combining several local horizontal barriers. In [32,33], the authors proposed the concepts of an event-driven partial barrier and a reinforced barrier. These two kinds of barriers can detect movements in various directions, which enhances the monitoring capability of the conventional sensor barrier. The ideas of these barriers resemble ours in that we both intend to strengthen barrier coverage by building complex barriers with more than one pair of start and end points. Nevertheless, in this paper, we utilize and evaluate more methods of selecting appropriate sensor nodes in order to build the maximum number of crossed barriers.

Thus, inheriting the idea of using the barriers in both horizontal and vertical directions to reinforce the robustness of a network, we consider introducing crossed barriers as a substitute for conventional barriers to strengthen the monitoring capability of a single barrier. We will further explain the detailed models and problem formulations in the following sections.

## 3. Models and Problem Statement

We consider a WSN with *n* omni-directional sensors $S=\{s_{1},s_{2},\ldots ,s_{n}\}$ randomly deployed to monitor a rectangular region *B* with length $L_{x}$ and width $L_{y}$.

**Figure 2:**
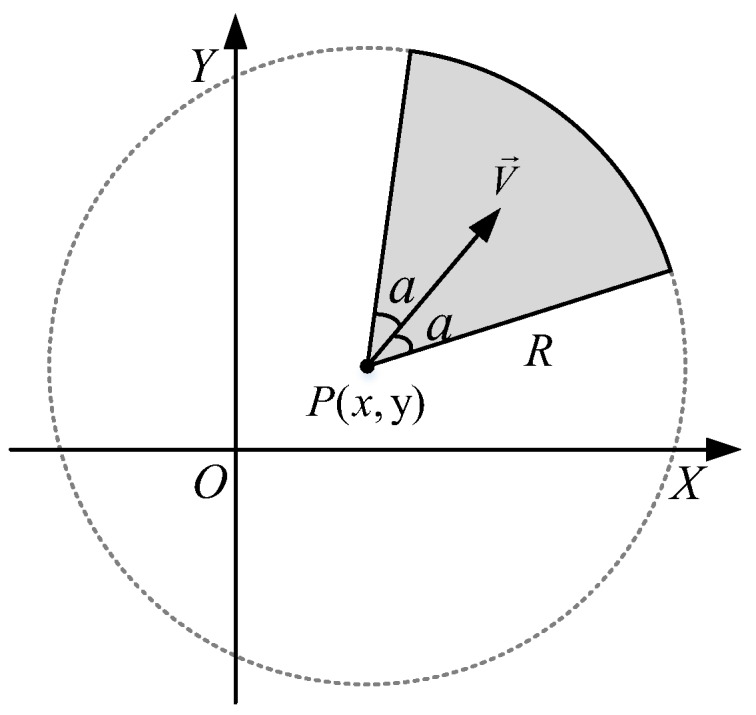
The quadruple sensing model in a WSN.

As shown in Figure 2, we represent a senor node with a sector denoted by a quadruple $s_{i}=\langle P_{i},R,a,{\vec{V}}_{i}\rangle$, where $P_{i}$ is the coordinate of the sensor in a two-dimensional monitoring plane; *R* is the maximum sensing radius and *a* is the sensing offset angle; ${\vec{V}}_{i}$ is a unit vector, representing the sensing orientation of the node. The shaded sector shown in Figure 2 represents the field of view (FoV) of a sensor node. In addition, the Boolean sensing model [23] is used. In this model, all the space points within the FoV can be detected with possibility one and can be said to be covered by the sensor. The points outside the FoV have no possibility of being detected and cannot be said to be covered by the sensor.

The conventional omni-directional sensing model is a special case of the quadruple sensing model when a=π and $\vec{V}$ is an arbitrary unit vector. Note that we adopt this model since it can also be used in applications of DSNs where the directional sensor is modeled; however, in the following analysis and simulations, we continue to use the omni-directional sensing model for simplicity. Thus, we introduce some definitions here to better describe the problem.

**Definition** **1.** **Strong barrier**. A strong barrier is a continuous horizontal or vertical path that consists of a series of sensor nodes whose sensing regions overlap with adjacent ones and guarantee detection of intruding paths in both vertical and horizontal directions.

**Definition** **2.** **Crossed barrier**. A crossed barrier consists of both a vertical strong barrier and a horizontal one that do not share a common sensor, ensuring detection of any intruding path in both vertical and horizontal directions.

**Definition** **3.** **Directional coverage graph** G(V,E). A directional coverage graph of a wireless sensor network *S* is constructed as follows: *G* represents the graph made up by the sets of vertexes *V* and directed edges *E*. The set *V* consists of vertexes corresponding to the sensors in the network. In addition, *V* has four virtual nodes, s1, t1, s2 and t2, that correspond to the left, right, upper, and lower boundaries, respectively. A directed edge exists between two nodes if their sensing regions overlap in the deployment region *B*. The direction of the edge is from the node that is closer to s1 (or s2) to the node closer to t1 (or t2). An edge exists between a vertex and a particular virtual node if the sensing region of the corresponding sensor overlaps with a boundary of the region.

**Figure 3:**
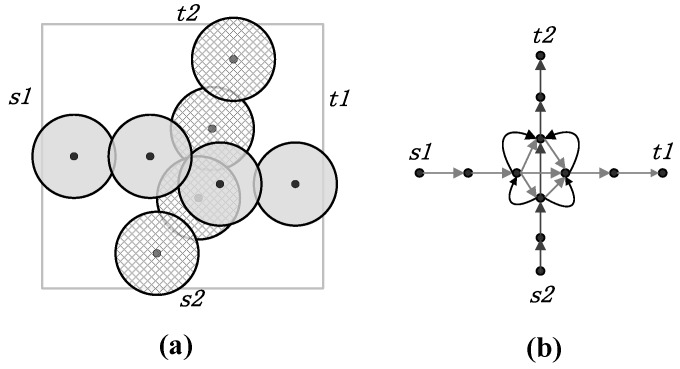
An illustrations of a crossed barrier and a directional coverage graph. (**a**) A crossed barrier. (**b**) The directional coverage graph showing the overlapping relation between nodes.

An illustration of crossed barrier coverage and a directional coverage graph is presented in Figure 3. As shown in Figure 3a, the horizontal strong barrier, which connects the leftmost border s1 and the rightmost border t1 of the surveillance region, and the vertical one, which connects s2 and t2, together constitute a crossed barrier. In Figure 3b, a directional coverage graph is given to model the network shown in Figure 3a. The dots represent sensor nodes or boundaries, while the directed edges with arrows show the overlapping relationship between sensor nodes. Since we always build barriers from particular sides of a network for convenience, we choose s1 and s2 as the start points of building horizontal and vertical barriers. The direction of an edge indicates the relative order of two connected nodes. The graph in the context of graph theory provides a clear description of network topology.

In the coverage graph of Figure 3b, if we can find two paths that link (s1, t1) and (s2, t2), respectively, and are disjoint with respect to each other, we can easily obtain a crossed barrier in Figure 3a. In fact, [7,14] provide a method for finding node-disjoint paths. However, since there are two pairs of virtual nodes in our problem, the previous method cannot be directly applied.

The main target of our research is to find the maximum number of crossed strong barriers, since scheduling the sensor barriers to work alternately can efficiently prolong network lifetime. Thus, we need to find more pairs of disjoint paths in the corresponding directional coverage graph. The following theorem is presented to analyze the computational complexity.

**Theorem** **1.** The problem of finding the maximum number of crossed barriers is NP-hard.

**Proof** **of** **Theorem** **1.** By definition, a crossed barrier is made up of two barriers that do not have common sensors and lie in perpendicular directions. From the directional coverage graph, we need to figure out the maximum pairs of disjoint horizontal and vertical paths. Thus, we turn the problem into a flow problem in graph theory to solve it.

We construct a new auxiliary graph to assist the proof. Every vertex $v_{i}$ (except for the four virtual nodes) in the directional graph is divided into two sub-vertexes $v_{i}^{in}$ and $v_{i}^{out}$. The inward edges go into $v_{i}^{in}$ and the outward ones come from $v_{i}^{out}$. There are inner edges linking the sub-vertexes with capacity set to one. The capacity of other edges is also set to one. The transformed directional coverage graph is equivalent to the previous one since every vertex should only be used once, as we have mentioned.

Assuming that the maximum number of crossed barriers is $R_{c}\in Z^{+}$, then the decision version of the problem can turn into the directed two-commodity integral flow problem in [34] with $R_{1}=\;R_{2}=\;R_{c}$, where $R_{1}$ and $R_{2}$ are the flow from $s_{1}$ to $t_{1}$ and from $s_{2}$ to $t_{2}$. Since the equivalent directed two-commodity integral flow problem is NP-complete, then the optimization version of our problem, which is finding the maximum number of crossed barriers, is NP-hard. ☐

## 4. ILP Formulation & the Branch-and-Bound Method

In this section, we present an integer linear programming formulation whose solution will serve as a benchmark, and we use the branch-and-bound algorithm to solve it.

### 4.1. ILP Formulation

Based on the directional coverage graph, we develop an ILP formulation for our problem. In the following formulation, $f_{e_{i}}^{hor}$ and $f_{e_{i}}^{ver}$ represent the flow on edge $e_{i}$, where subscripts hor and ver indicate that a flow is a part of the horizontal or vertical paths; $E_{v_{j}}^{in}$ and $E_{v_{j}}^{out}$ stand for the sets of inwards and outward edges of vertex $v_{j}$ in *V*.

(1) $$\max\;\min\;(\sum_{e_{i}\in E_{t1}^{in}}f_{e_{i}}^{hor},\sum_{e_{i}\in E_{t2}^{in}}f_{e_{i}}^{ver})$$

s.t.

(2) $$\begin{array}{cc} \sum_{e_{i}\in E_{v_{j}}^{in}}(f_{e_{i}}^{hor} & +f_{e_{i}}^{ver})=\sum_{e_{i}\in E_{v_{j}}^{out}}\left(f_{e_{i}}^{hor}+f_{e_{i}}^{ver}\right)\;,\;\forall v_{j}\in V\setminus \left\{s1,t1,s2,t2\right\} \end{array}$$

(3) $$\sum_{e_{i}\in E_{v_{j}}^{in}}\left(f_{e_{i}}^{hor}+f_{e_{i}}^{ver}\right)\leq 1\;,\;\forall v_{j}\in V\setminus \left\{s1,t1,s2,t2\right\}\;$$

(4) $$\left(f_{e_{i}}^{hor}+f_{e_{i}}^{ver}\right)\leq 1\;,\;\forall e_{i}\in E$$

(5) $$f_{e_{i}}^{hor},f_{e_{i}}^{ver}=\left\{0,1\right\}.$$

To be specific, Equation (1) concerns finding the maximum number of crossed barriers. Since a crossed barrier constitutes barriers in two directions, the optimization target is maximizing the minimum value of the barrier numbers in horizontal and vertical directions, rather than maximizing the barrier number in a single direction. Equation (2) is a flow conservation equation, which arises from the fact that the flows on the inward edges of a vertex (except for s1, s2, t1 and t2) increases equally with those on the outward edges. Furthermore, with the requirement of getting disjoint paths in the directional coverage graph, Equation (3) guarantees that the maximum time a sensor node can be used are always bounded by one. Finally, Equations (4) and (5) ensures that the maximum time an edge can be used are less than one.

However, it seems that Equation (1) is not in the standard form of ILP, which means we cannot use general methods to analyze and solve it. Thus, some further work is done here to transform object function into a standard one. We introduce an integer $f_{c}\in Z^{+}$, which satisfies the following inequations:

(6) $$f_{c}\leq \sum_{e_{i}\in E_{t1}^{in}}f_{e_{i}}^{hor}$$

(7) $$f_{c}\leq \sum_{e_{i}\in E_{t2}^{in}}f_{e_{i}}^{ver}.$$

Then, Equation (1) can be replaced by the following function:

(8) $$\max\;f_{c}.$$

Here, $f_{c}$ can be seen as the minimum value of the flows in two directions. Therefore, Equation (1) is replaced by Equation (8) with two extra conditions, Equations (6) and (7), added to guarantee that $f_{c}$ is not bigger than the flow value in any direction.

### 4.2. The Branch-and-Bound Method

Since finding the maximum number of crossed barriers is NP-hard, there is no algorithm that can obtain the optimal solutions in polynomial time. We have the ILP formulation for the problem, so a simple idea is to use brute-force enumeration, which involves systematically enumerating all possible solutions and checks whether each candidate satisfies the problem’s statement. However, the exponential growth of effort required to examine all possible candidates is unacceptable for a WSN with many nodes. Thus, we consider solving it using the branch-and-bound algorithm [35,36], which uses selective enumeration rather than complete enumeration.

For the branch-and-bound algorithm, there are two essential components: branching and bounding. Branching is the operation that divides a problem into two or more subproblems such that the solution of the original problem can be achieved from the solutions of the subproblems. For the above ILP, a simple branching operation is to pick a variable ($f_{e_{i}}^{ver}$ or $f_{e_{i}}^{hor}$) and to replace the current problem with two subproblems. Thus, both subproblems will be copies of the current problem, where the variable ($f_{e_{i}}^{ver}$ or $f_{e_{i}}^{hor}$) is set to 0 in one copy and set to 1 in the other. Since the variable ($f_{e_{i}}^{ver}$ or $f_{e_{i}}^{hor}$) has to take either 0 or 1 in an optimal solution, the branching scheme guarantees that an optimal solution of the original problem will be an optimal solution of one of the two subproblems.

Bounding is a function that returns a bound on the optimal solution of the current subproblem. For the maximization problem, the returned value will be a number larger or equal to an optimal solution. For our problem, a simple choice is to remove the integrality constraints on the variables by replacing them with lower and upper bounds on the variables, and to transform the subproblem to a linear programming (LP) problem. This operation is called the LP relaxation of the subproblem, which is quite easy to solve. Since the feasible solutions of the ILP subproblem are all feasible for the LP problem, the optimal solution of the former will always be worse or equal to the optimal solution of the latter. Moreover, an additional use of the bound gained is that it is possible to discard subproblems that have a bound value worse than the value of the best currently known solution of the original problem.

Although the worst-case complexity of the branch-and-bound algorithm is still an exponential time algorithm, it can speed up the calculation for average cases.

## 5. Proposed Algorithms

In this section, we present a multi-round shortest path algorithm (MSPA), which works heuristically to guarantee efficiency while maintaining good solutions. Heuristic methods are widely used to solve different coverage problems, e.g., barrier coverage in [31] and target coverage in [37], to improve calculation efficiency. In addition, for the purpose of comparison, some conventional algorithms of finding maximum strong barriers are also modified here to adjust to the new problem.

### 5.1. The Multi-Round Shortest Path Algorithm (MSPA)

As mentioned before, the horizontal strong barrier should be vertex-disjoint or sensor-disjoint with a vertical one to constitute a crossed barrier. Thus, we propose choosing the sub-barrier alternately and removing nodes on a sub-barrier from the directional coverage graph G(V,E) once the sub-barrier is chosen. The pseudo code of MSPA is presented in Algorithm 1.

In the pseudo code, Dijkstra(G,vertex1,vertex2) represents the function to calculate the shortest path from vertex1 to vertex2 in *G* using Dijkstra’s algorithm [38], and Remove(G,vertex_set) is a function which removes the vertexes and edges related to the vertexes in vertex_set. Since the function Remove() is executed after a single-direction path is found, it ensures that all sub-barriers are node-disjoint with each other.

Dijkstra’s algorithm can find the shortest paths between nodes in a graph. In our algorithm, the length of a path is counted by the hop counts of sensor nodes on it. Since a path with the least hop counts may avoid using extra sensor nodes, we choose the horizontal and vertical barriers using the metric of hop counts.

The computational complexity of Dijkstra’s algorithm is O(|V|*log(|V|)+|E|), where |V| and |E| are the number of vertexes and edges, respectively. Since |V|=n and $\left|E\right|=n^{2}$ at most, the worst-case time complexity of the MSPA is $O(n^{2})$, which is significantly lower than that of the branch-and-bound algorithm.

**Algorithm 1:** The Multi-Round Shortest Path Algorithm**Input**: The directional coverage graph *G* of a WSN;**Output**:The maximum number of crossed barriers $N_{max}$;The set of nodes on the $i^{th}$ crossed barrier $Q_{i}$;**1**$i\leftarrow 0;\;N_{max}\leftarrow 0;Q_{hor}\leftarrow \emptyset ;Q_{ver}\leftarrow \emptyset$;**2****while**
*True*
**do****3** **if**
*there exist a path from s1 to t1 on G(V,E)*
**then****4**  $Q_{hor}\leftarrow$ Dijkstra(*G*,s1,t1);**5**  G← Remove(*G*, $Q_{hor}$);**6** **else****7**  break;**8** **end****9** **if**
*there exist a path from s2 to t2 on G(V,E)*
**then****10**  $Q_{ver}\leftarrow$ Dijkstra(*G*,s2,t2);**11**  G← Remove(*G*, $Q_{ver}$);**12** **else****13**  break;**14** **end****15** $N_{max}\leftarrow N_{max}+1$;**16** $i\leftarrow i+1;\;Q_{i}\leftarrow Q_{hor}\cup Q_{ver};$**17****end**

### 5.2. Max-Flow-Least-Conflicts Algorithm and Max-Flow-Least-Counts Algorithm

The maximum flow algorithm such as the Edmonds–Karp algorithm [39] can calculate the maximum flow of a network in polynomial time, and it is chosen in many literatures to figure out the maximum node–disjoint barriers. Taking advantage of the maximum flow algorithm, we present the max-flow-least-conflicts algorithm and the max-flow-least-counts algorithm in this section.

As an auxiliary graph is constructed when proofing Theorem 1, we can obtain the maximum numbers of disjoint strong barriers on horizontal and vertical directions individually by using the maximum flow algorithms to the auxiliary graph. Moreover, the horizontal (or vertical) strong barriers are node-disjoint with each other since the capacities for all the inner edges in the auxiliary graph are set to 1, which restricts the maximum time that sensor nodes can be used.

For the max-flow-least-conflicts algorithm, we present a step-by-step heuristic method to combine the horizontal and vertical barriers to make crossed barriers. The pseudo code is presented in Algorithm 2.

In each step, a horizontal (or vertical) path, which conflicts with the fewest number of the other paths in the vertical (or horizontal) direction, is selected, and the conflicting paths and the path selected are removed from the auxiliary graph. In Lines 6 and 17 of the pseudo-code, *M* is a matrix showing the conflicting relations between horizontal flows and vertical flows. The columns represent the horizontal flows, while the rows represent the vertical flows. An element in the matrix *M* is set to 1 when the corresponding flows in the two directions have common nodes. By searching the matrix, we can quickly determine whether a path conflicts with others. The path selection procedure is executed alternately between horizontal and vertical directions, and a pair of these operations leads to a crossed barrier.

**Algorithm 2:** The Max-Flow-Least-Conflicts Algorithm**Input**: The directional coverage graph *G* of a WSN;**Output**: The maximum number of crossed strong barriers $N_{max}$;The set of nodes on the $i^{th}$ crossed strong barrier $Q_{i}$;**1** Construct an auxiliary graph $G^{\prime }$ of *G*;**2** Calculate the sets of horizontal flows $F^{hor}$ through Maxflow($G^{\prime }$, s1, t1), and the sets of vertical flows $F^{ver}$ through Maxflow($G^{\prime }$, s2, t2);**3**$i\leftarrow 0;\;N_{max}\leftarrow 0;Q_{hor}\leftarrow \emptyset ;Q_{ver}\leftarrow \emptyset$;**4****while**
*True*
**do****5** **if**
$F^{hor}\neq \emptyset$
**then****6**  Calculate the conflicting matrix *M*, and pick a flow $f_{i}^{hor}$ in $F^{hor}$ with least conflicts in *M*;**7**  $Q_{hor}\leftarrow f_{i}^{hor};F^{con}\leftarrow$ the flows that conflict with $f_{i}^{hor}$;**8**  **if**
$F^{con}\subset \emptyset$
**then****9**   Remove $f_{i}^{hor}$ from $F^{hor}$;**10**  **else****11**   Remove $f_{i}^{hor}$ from $F^{hor}$ and $F^{con}$ from $F^{ver}$;**12**  **end****13** **else****14**  break;**15** **end****16** **if**
$F^{ver}\neq \emptyset$
**then****17**  Update the conflicting matrix *M*, and pick a flow $f_{i}^{ver}$ in $F^{ver}$ with least conflicts;**18**  $N_{max}\leftarrow N_{max}+1;i\leftarrow i+1;Q_{ver}\leftarrow f_{i}^{ver};\;Q_{i}\leftarrow Q_{hor}\cup Q_{ver}$;**19**  $F^{con}\leftarrow$ the flows that conflict with $f_{i}^{ver}$;**20**  **if**
$F^{con}\subset \emptyset$
**then****21**   Remove $f_{i}^{ver}$ from $F^{ver}$;**22**  **else****23**   Remove $f_{i}^{ver}$ from $F^{ver}$ and $F^{con}$ from $F^{hor}$;**24**  **end****25** **else****26**  break;**27** **end****28****end**

The basic idea of the max-flow-least-counts algorithm is similar to that of the max-flow-least-conflicts algorithm. However, the path selection procedure is different. In Lines 6 and 17, the max-flow-least-counts algorithm will choose the flows with the least hop counts. The pseudo-code for the this algorithm is omitted to save space.

For a network with *n* sensor nodes, there are 2n+4 nodes in their auxiliary graph. The computational complexity of using the Edmonds-Karp algorithm is $O(|V|\times |E{|}^{2})$, where |V| and |E| are the number of vertexes and edges, respectively. Thus, the worst-case computational complexity of getting the maximum flows by using the Edmonds-Karp algorithm is $O(n^{3})$. For the operation in calculating the conflicting matrix, the worst-case complexity is $O\left(\left(2n+4\right)\times \left(\left(2n+4\right)-1\right)/2\right)=\;O\left(n^{2}\right)$. Thus, the worst-case computational complexities for both the max-flow-least-conflicts algorithm and the max-flow-least-counts algorithm are $O(n^{3})$-slightly higher than that of the MSPA.

Overall, the idea of choosing the flows with the least conflicts or counts is similar to the work in [31]. However, we have modified their rules of choosing vertex-disjoint paths with a single source–sink pair so that the new ones can fit in with the crossed barrier problem, where paths from two path groups that have different sources and sinks need to be chosen.

**Algorithm 3:** The Max-Flow-Maximum-Independent-Set Algorithm
**Input**: The directional coverage graph *G* of a WSN;**Output**:The maximum number of crossed strong barriers $N_{max}$;The set of crossed strong barrier *Q*;**1**Construct an auxiliary graph $G^{\prime }$ of *G*;**2** Calculate the sets of horizontal flows $F^{hor}$ through Maxflow($G^{\prime }$, s1, t1), and the sets of vertical flows $F^{ver}$ through Maxflow($G^{\prime }$, s2, t2);**3** Calculate the conflicting matrix *M* for $F^{hor}$ and $F^{ver}$;**4** Combine the non-conflict barriers in $F^{hor}$ and $F^{ver}$ to obtain the set of potential crossed barriers $F^{pon}$;**5** Construct an auxiliary graph $G^{\prime \prime }$ for $F^{pon}$;**6***Q* ← MaxISalgorithm($G^{\prime \prime }$);**7**$N_{max}$ ← |Q|;

### 5.3. The Max-Flow-Maximum-Independent-Set Algorithm

In the two algorithms above (Algorithms 2 and 3), barriers from the sets of horizontal and vertical barriers are chosen to build a crossed barrier on the basis of the number of conflicts or hop counts. In this section, we consider selecting proper sub-barriers by introducing the concept of a maximum independent set.

In graph theory, an independent set (IS) is a set of vertexes in a graph where no two vertexes are adjacent. To be specific, it is a set $S_{v}$ of vertexes such that, for every two vertexes in $S_{v}$, there is no edge connecting them. A maximal independent set (MIS) is an independent set that is not a subset of any other independent set. In other words, there is no vertex outside the independent set that may be added, since it is maximal with respect to the independent set property. A maximum independent set (MaxIS) is a MIS with maximum cardinality in a graph.

**Figure 4:**
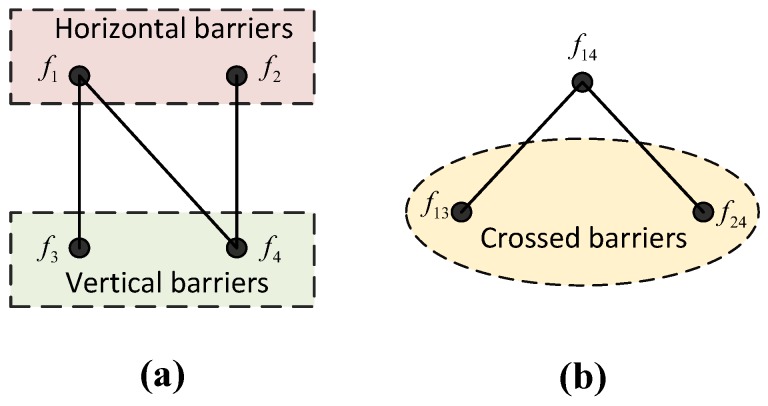
The illustrations of the max-flow-maximum-independent-set algorithm. (**a**) The barriers in individual directions. (**b**) The maximum independent set (MaxIS) of the graph is calculated to obtain the maximum crossed barriers.

Since we can obtain the maximum number of node-disjoint barriers in horizontal and vertical directions, we consider combining the non-conflict barriers to make potential crossed barriers and then utilize the algorithms of calculating the MaxIS to obtain a set of crossed barriers that has maximum cardinality and no conflict. We present a simple example in Figure 4 to illustrate how the max-flow-maximum-independent-set algorithm works.

In Figure 4, $f_{1}$ and $f_{2}$ are the node-disjoint barriers in the horizontal direction, which is calculated by the maximum flow algorithm, while $f_{3}$ and $f_{4}$ are those in the vertical direction. If $f_{2}$ conflicts with $f_{3}$, which means that $f_{2}$ has one or more common nodes with $f_{3}$, we can obtain the relations shown in Figure 4a, where the edges in the graph indicate that the barriers they have linked can be combined together to constitute a crossed barrier. Thus, we can obtain three potential crossed barriers: $f_{13}$, $f_{14}$ and $f_{24}$. An auxiliary graph as shown in Figure 4b is then constructed in which the vertexes represent potential crossed barriers and the edges indicate that there are conflicts between vertexes. For our example, since $f_{14}$ shares the barriers with $f_{13}$ and $f_{24}$, by utilizing a heuristic algorithm [40], we can obtain the maximum independent set of the graph, which includes $f_{13}$ and $f_{24}$. Consequently, we can obtain the crossed barriers $f_{13}$ and $f_{24}$, and the maximum number of crossed barriers is two.

The core purpose of introducing the MaxIS is to model the conflicting relations in real world applications in a virtual graph and then use heuristic algorithms to handle it. In an auxiliary graph, two conflicting barriers can be mapped to two vertexes with an edge linking them. Thus, when using the algorithms for MaxIS, the two vertexes cannot be in an independent set at the same time with respect to the independent set property.

We present the pseudo-code for the max-flow-maximum-independent-set algorithm in Algorithm 3. In Step 6, MaxISalgorithm($G^{\prime \prime }$) is a function to obtain the maximum independent set by using the heuristic algorithm called the vertex support algorithm (VSA) [40]. The time complexity for Steps 1 to 2 is $O(n^{3})$. For Steps 3, 4, and 5, the worst-case time complexity is $O(n^{2})$. Therefore, the time complexity of the max-flow-maximum-independent-set algorithm is $O(n^{3})$. The worst-case computational complexities for it are not as good as that of MSPA but still outperform those of the branch-and-bound algorithm.

Furthermore, the connectivity issue of sensor nodes is already taken into account in all these three algorithms, since, when barriers are built, adjacent nodes need to able to communicate and have overlapped sensing areas with each other.

## 6. Evaluation

In this section, we evaluate the performance of the proposed algorithms via extensive simulations in terms of the number of crossed barriers. The purpose of the simulations is twofold: to prove the effectiveness of the MSPA in comparison with other algorithms when considering both time complexity and solution accuracy, and to evaluate the effect of important parameters (such as the size of target region, the number of sensor nodes, the sensing radius, and the sensing offset angle) on the crossed barrier number. The following simulation experiments were performed on a laptop computer equipped with an Intel(R) Core(TM) i7-4700MQ processor, an 8-GB memory, and the 64-bit Windows 10 operating system. The simulation codes are based on the Matlab language.

Assume that, for definiteness and without loss of generality, omni-directional sensors are uniformly deployed in a square region with dimensions of 150×150 m. A didactic example is included in Scenario 1 to illustrate the algorithms.

**Figure 5:**
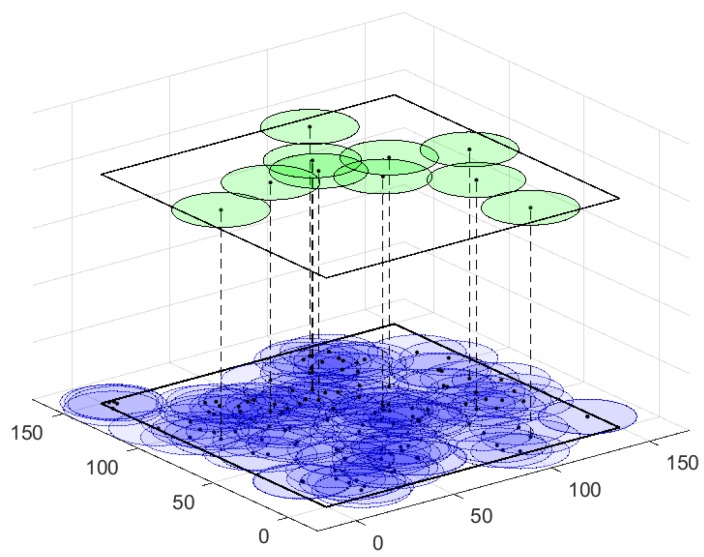
Scenario 1: The deployment map of a WSN and a crossed barrier calculated by the MSPA.

In Scenario 1, R=40 m and n=120. As shown in Figure 5, the deployment map shows the locations of sensor nodes in the square region. The areas in dark blue indicate that they are well monitored or covered by sensor nodes and that some sensors may be redundant. In order to achieve crossed barriers in the network, branch-and-bound, MSPA, and other proposed algorithms are executed to compare the maximum number of crossed barriers they can obtain.

First, one of the crossed barriers calculated by the MSPA is illustrated in Figure 5. The sensor nodes on the barrier, represented by the green circles, form two continuous strong barriers linking two pairs of boundaries that can further constitute a crossed barrier. Thus, our MSPA can identify crossed barriers correctly from the network.

Then, we present the crossed barrier numbers calculated by different algorithms. The branch-and-bound algorithm (B&B for short) can obtain 10 crossed barriers, which is optimal among these algorithms. For the MSPA, the max-flow-least-conflicts algorithm (least-conflicts, for short), the max-flow-least-counts algorithm (least-counts for short), and the max-flow-maximum-independent-set algorithm (MaxIS for short), the maximum numbers of crossed barriers are 6, 4, 4, and 5, respectively. Although B&B outperforms MSPA in the number of crossed barriers, the MSPA still achieves a good solution, with the worst-case time complexity being kept at a reasonable level. For a network with a substantial number of sensor nodes, time complexity needs to be taken into consideration. Using heuristic algorithms are important for balancing the precision of solutions and the computation complexity.

In the following scenarios, we mainly conduct simulations to evaluate the effects of node number (*n*), sensing radius (*R*), and sensing offset angle (*a*) on the maximum number of crossed barriers by setting them to different values. All simulations are done in two configurations, where the sizes of the target region are set to 150×150 m and 150×75 m, respectively, and the results are the statistical average of 100 simulations.

**Figure 6:**
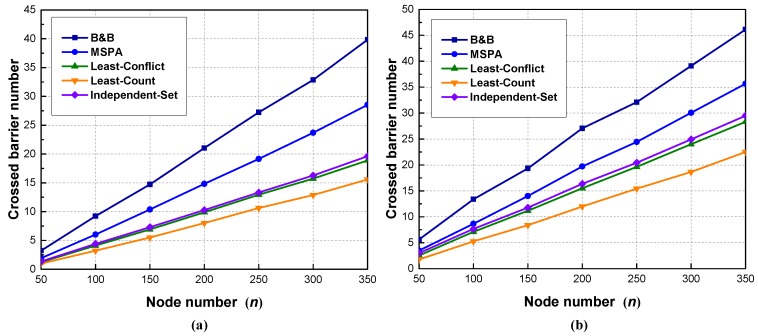
Scenario 2: The effect of node number on crossed barrier number: (**a**) region size: 150×150 m; (**b**) region size: 150×75 m.

In Scenario 2, R=20 m and *n* ranges from 50 to 350, with 50 as the increment. As can be observed in Figure 6, the crossed barrier numbers of all the algorithms increase monotonically and linearly with the increase in node number. This is because deploying more sensor nodes in a fixed-area region leads to a higher density for sensor nodes, which in turn helps to enhance the connectivity of sensor nodes and provides more paths for constructing crossed barriers. Additionally, the number of crossed barriers in Figure 6b is higher than that in Figure 6a with the same node number, which is also related to the density of the sensor nodes, rather than the size of the region.

B&B provides optimal solutions that will serve as benchmarks for other algorithms. The MSPA outperforms other proposed algorithms but achieves a sub-optimal solution. Considering, however, that B&B is an enumeration method while MSPA is a heuristic one, the performance of the MSPA is acceptable. Furthermore, the MSPA has better performance than the other three algorithms in which the maximum flow algorithm is used to calculate the sub-barriers. The MSPA identifies more crossed barriers with lower computational complexity. Thus, the operation of deciding the sub-barriers first may limit the maximum number of crossed barriers. In addition, the independent-set performs better than the least-conflicts and the least-counts, since, after determining the maximum flows, the independent-set provides more combinations of sub-barriers. Least-counts yields the worst results since the hop counts may be too simple to be a good indicator of which path should be chosen.

**Figure 7:**
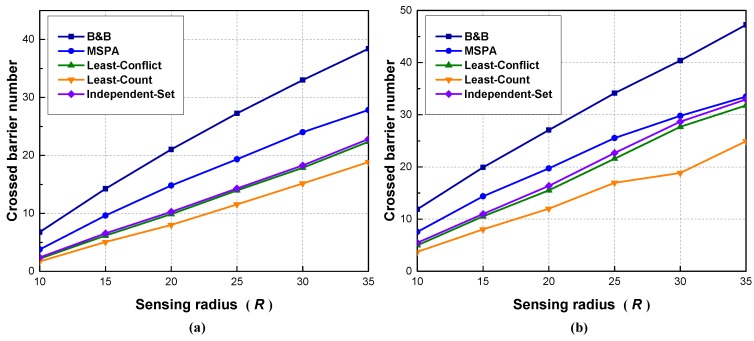
Scenario 3: The effect of sensing radius on crossed barrier number: (**a**) region size: 150×150 m; (**b**) region size: 150×75 m.

In Scenario 3, n=200 and the sensing radius *R* is changed from 10 to 35 m with 5 m as the increment. Observing Figure 7, we can obtain the similar monotonically increasing trend as that in Scenario 2. However, in Figure 7b, the solutions of the MSPA, the least-conflicts, and the least-counts become closer as sensing radius increases. The reason for this phenomenon is that there are limitations to the solutions of all five algorithms when the sensing radius reaches a relatively high value. Considering an extreme case in which *R* reaches a value such that all the sensors can cover the whole region, then there is no use of increasing the maximum number of crossed barriers when increasing *R*. The solutions of all algorithms will approach their limit when *R* is high enough. Additionally, the MSPA has a better performance than other algorithms.

In Scenario 4, we consider changing the sensing offset angle *a* to evaluate its effect. Since there are many applications related to utilizing a directional sensing model instead of an omni-directional one, the simulations may attest to the effectiveness of algorithms using different sensing models.

**Figure 8:**
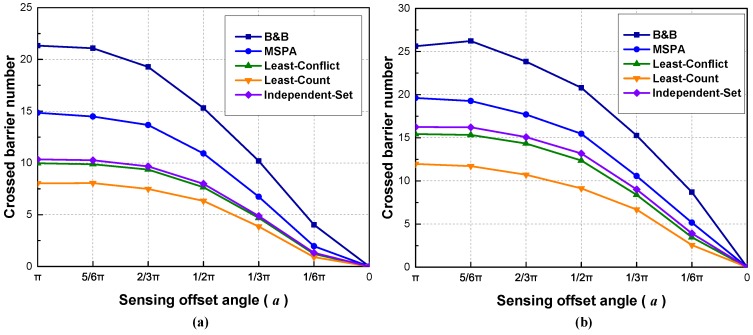
Scenario 4: The effect of sensing offset angle on crossed barrier number: (**a**) region size: 150×150 m; (**b**) region size: 150×75 m.

In this scenario, n=200, R=20 m, and *a* is changed from π to 0 with a decrement of 1/6π. A random unit vector is assigned to each sensor node as the sensing orientation. As shown in Figure 8, all algorithms have a downward trend when decreasing the sensing offset angle. The reason for this is that, with the limitation in the sensing offset angle, the area that a sensor node can cover becomes smaller, which consequently leads to a lower chance for sensor nodes to have an overlapped sensing area and form barriers. We have noted that, in Figure 8a, when *a* is close to π or 0, the slopes of the curves are relatively gentle. The probability of two sensor nodes to have overlapped sensing regions becomes either too high or too low when *a* is close to π and 0. Thus, the curves in the figure have different slopes. In Figure 8b, the slope between 1/6π and 0 is still substantial. The reason lies in the fact that a greater density of sensor nodes may slow down convergency. When deploying directional sensors to achieve crossed barriers, we may need to increase the number of sensor nodes to achieve an adequate number of crossed barriers.

Through the simulations above, we evaluated the effect of some important parameters on crossed barrier coverage. We found that all parameters have a direct effect on the coverage intensity of the entire region, which will further affect the maximum number of crossed barriers that can be found.

## 7. Conclusions

We herein studied the problem of achieving the maximum number of crossed barriers in a wireless sensor network. We first defined a crossed barrier, which can overcome the defect of traversal intrusion, and presented the problem of maximizing the number of crossed barrier. Then, we proved that the problem is NP-hard in computation complexity. We further developed the ILP formulation for the optimization problem and used the branch-and-bound algorithm to obtain an optimal solution. Moreover, we proposed an efficient MSPA algorithm along with some other heuristic algorithms. Through theoretical analysis and simulations, we found that the MSPA outperforms other algorithms in both the worst-case computational complexity and the maximum number of crossed barriers achieved.
